# Demethylation of ITGAV accelerates osteogenic differentiation in a blast-induced heterotopic ossification *in vitro* cell culture model

**DOI:** 10.1016/j.bone.2018.09.008

**Published:** 2018-12

**Authors:** Niall J. Logan, Marie Camman, Greg Williams, Claire A. Higgins

**Affiliations:** a Department of Bioengineering, Imperial College London, London SW7 2AZ, United Kingdom,; b Farjo Hair Institute, London, W1G 7LH, United Kingdom

**Keywords:** Epigenetic DNA methylation, Heterotopic ossification, *In vitro* model, Blast overpressure exposure, Ossification

## Abstract

Trauma-induced heterotopic ossification is an intriguing phenomenon involving the inappropriate ossification of soft tissues within the body such as the muscle and ligaments. This inappropriate formation of bone is highly prevalent in those affected by blast injuries. Here, we developed a simplified cell culture model to evaluate the molecular events involved in heterotopic ossification onset that arise from the shock wave component of the disease. We exposed three subtypes of human mesenchymal cells *in vitro* to a single, high-energy shock wave and observed increased transcription in the osteogenic master regulators, Runx2 and Dlx5, and significantly accelerated cell mineralisation. Reduced representation bisulfite sequencing revealed that the shock wave altered methylation of gene promoters, leading to opposing changes in gene expression. Using a drug to target ITGAV, whose expression was perturbed by the shock wave, we found that we could abrogate the deposition of mineral in our model. These findings show how new therapeutics for the treatment of heterotopic ossification can be identified using cell culture models.

## Highlights

• Development of an *in vitro* cell culture model to study blast-induced heterotopic ossification
• Application of a single shock wave in air leads to enhanced osteogenic response in cells.
• Shock wave exposure results in demethylation of the ITGAV promoter.
• Inhibition of ITGAV using cilengitide can abrogate mineral deposition within our *in vitro* model.

## Graphical abstract

**Figure:**
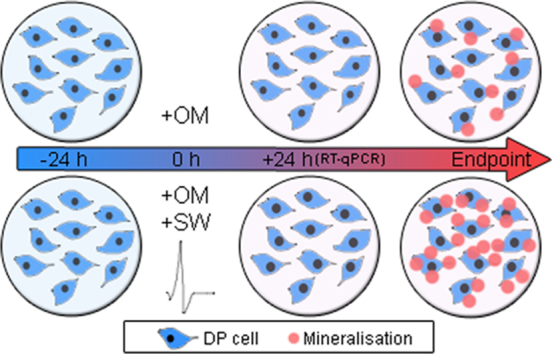

## 1 Introduction

Heterotopic ossification (HO) is a form of inappropriate ossification that results in the formation of mature ectopic bone within soft tissues of the body, including muscle, tendons and ligaments. This calcification of soft tissue can result from genetic mutations that cause rare diseases such as fibrodysplasia ossificans progressiva [[1], [2], [3]] or progressive osseous heteroplasia [3,4], although intriguingly it is more common following high energy injuries or trauma. These can include traumatic brain injury [[5], [6], [7]], spinal cord injury [6,8], total arthroplasty procedures [9], fractures [[10], [11], [12]] and burns [13]. Additionally, one of the most prevalent HO-inducing injuries are extremity wounds obtained through exposure to blast events. In such cases, the incidence of HO can be as high as 63% when the mechanism of injury is a single high energy shock wave [14,15]. Current methods of prophylaxis, such as non-steroidal anti-inflammatory drugs [10,[16], [17], [18], [19]] and radiotherapy [18,20], can reduce the incidence of trauma-induced HO, but are by no means a cure for the disease [21,22]. Thus, there is a need to develop model systems capable of breaking down the individual components causative of trauma-induced HO, to study their specific roles in disease onset, so that we can identify new therapeutics to prevent HO.

One difficulty in understanding how trauma can result in HO lies in the complexity of the disease. Animal models for trauma-induced HO have been developed [23], although there is only one rodent model which specifically looks at air-driven blast-induced HO, and this recreates several aspects of the trauma, from the blast through to the extremity injury and subsequent amputation [[24], [25], [26]]. While this is advantageous to assess a whole body systemic response, and that of several cell types proposed to be involved in HO, it is impractical to use to determine the effect of individual cells to specific aspects of the trauma, such as the shock wave alone. Analysis of serum from patients has also revealed that there is a systemic response following injury [27], which is likely associated with HO onset as wound effluent from extremity wounds following blast can accelerate osteogenic differentiation of mesenchymal stem cells in culture [28]. However, none of these models above enable conclusive assessment of the effect of a single shock wave alone, representative of a blast event. Here, we wanted to develop a simple cell culture model system which would enable us to separate out the effects of the systemic response instigated by injury, from the shock wave which causes injury. We specifically set out to assess the response of cells in culture to a single high-energy shock wave.

Another challenge for the development of preventative treatments for HO is the diverse population of cell types thought to be responsible for ectopic bone lesions [[29], [30], [31], [32], [33], [34]]. However, one benefit of a cell culture model system is that the cell type responsible for HO *in vivo* need not be used. We propose that the ideal cell type to study HO *in vitro* should have the osteogenic capacity to differentiate into bone, but not do so under normal growth conditions, and thus be representative of inappropriate ossification. Cell types such as bone marrow mesenchymal stem cells (BM-MSCs), have been widely used to study osteogenesis, and even HO [28], as these cells may be involved in HO *in vivo*. However, because they readily facilitate bone formation [35], they may not provide a true representation of ectopic or inappropriate bone formation in an *in vitro* model. Human dermal papilla (DP) cells are specialised mesenchymal cells found at the base of the hair follicle that play a key role in hair growth and cycling [[36], [37], [38]], and are accordingly unrelated to bone. Curiously, while not being stem cells, human DP cells do have multipotent tendencies *in vitro* and can differentiate down both osteogenic and adipogenic lineages when grown in specific differentiation medias [[39], [40], [41]]. While differentiation capacity alone does not confer a large advantage over using BM-MSCs, DP cells also share a common developmental progenitor with papillary dermal fibroblasts (PFi) [42], also found in the skin. Despite arising from the same predecessor cell in development, PFi do not have the same differentiation capacity as DP [43] and do not mineralise *in vitro*, providing us with a unique biological sister cell type that can be used as a negative control when developing a cell culture model to investigate HO. While we do not believe DP cells play any role in HO *in vivo*, their inappropriate osteogenic differentiation potential and unique negative control sister cell type make them an ideal cell choice to develop a model of inappropriate bone formation, or HO, *in vitro*.

A second parameter to be taken into consideration in the study of trauma-induced HO is the type of shock wave that is used. It has long been reported that therapeutic shock waves in the form of extracorporeal shock wave (ESW) therapy can promote bone fracture healing [44] while repeated stimulus with thousands of shock waves, each in the mPa range, has been shown to enhance human BM-MSCs mineralisation *in vitro* [45,46]. However, while this form of mechanical perturbation is repetitive and long lasting in total duration, it is dissimilar to the shock wave created by an open field blast which last a few milliseconds in air, with peak pressures in excess of 100 kPa. Thus, in our study we chose to assess the effect of a single high-energy shock wave in air on cell differentiation, with the aim to specifically identify the role of the shock wave component in trauma-induced HO.

In cells such as DP and BM-MSCs, which can differentiate down both osteogenic and adipogenic lineages, there are a variety of factors which can sway the delicate balance, and impact differentiation direction [47]. For trauma-induced HO, we believe that the shock wave itself has an exacerbating role. We hypothesise that the shock wave transitions certain cells into a post shock state, where they become sensitised towards an osteogenic identity. In tandem, we already know that the injury caused by the blast results in an increase in osteogenic factors in the wound environment, which can also promote ossification [28]. In order to test our hypothesis, in this study, we established a cell culture model to study trauma-induced HO, separating out the effect of the shock wave from systemic influence. We used DP cells to evaluate inappropriate differentiation, and a compressed air-driven shock tube to create a single high-energy shockwave, in the kPa range. Surprisingly, the shock wave alone had no effect on cells, however, when cells were grown in osteogenic medium, we see a synergistic effect of the shock wave together with the medium, with accelerated and increased osteogenic differentiation and mineralisation in DP cells, compared to cells in osteogenic medium alone. We found that nuclear and epigenetic changes are occurring 6 h post shock wave exposure and chose this time point to perform reduced representation bisulfite sequencing (RRBS), to discover if changes to the methylation profile could help explain the accelerated osteogenesis observed in our cell model. We find that a single shock wave can alter the methylation profile of gene promoters, which leads to opposing transcriptional changes. Further, analysis of the hypomethylated promoters enabled identification of a mechanosensor, Integrin Alpha V (ITGAV), whose transcript increased in expression 24 h after shock wave exposure. Using cilengitide, a cyclic RGD pentapeptide [48], to block binding to and activation of ITGAV, we are able to rescind the accelerated osteogenic differentiation induced by shock wave exposure previously observed in our cell culture model. Thus we demonstrate that our cell culture model is suitable for the identification of therapeutics which can mitigate against the effect of the shock wave, and may be able to prevent HO onset after blast trauma.

## 2 Materials and methods

### 2.1 Study design

Here, we used primarily human DP cells, as well as other cells including human BM-MSC, human PFI and rodent DP cells, to create an *in vitro* cell culture model of blast trauma-induced HO, with the aim of identifying new targets for therapeutic intervention. We performed our experiment in four stages: (i) Firstly, we identified a non-lethal blast using live/dead imaging and Alamarblue assay, and using this 165 kPa shock wave we observed increased osteogenic differentiation in cells exposed to the shock wave in osteogenic media (OM + SW). Methods to study osteogenic differentiation included calcium assays, alizarin red staining and RT-qPCR; (ii) we next used immunofluorescence imaging to identify an early time point when changes to nuclear architecture were occurring in the OM + SW cells; (iii) using the 6 h time point identified in section (ii), we performed RRBS and generated differential lists where the methylation profile of a gene promoter had been altered by shock wave exposure. We validated our RRBS results by performing RT-qPCR and bisulfite sequencing, on selected genes with hyper- and hypo-methylated gene promoters; (iv) of the differentially methylated gene promoters in OM + SW, we identified ITGAV as a gene of interest and investigated its role in ossification. We found that treatment with cilengitide, which is an ITGAV inhibitor, could abrogate mineral deposition in our model. The sample sizes for our *in vitro* tests were taken from similar studies reported in the literature. The exact number for each experiment can be found in the figure legends. Investigators were not blinded when conducting or evaluating the experiments.

### 2.2 Cell isolation and culture

Human scalp skin biopsies were acquired as discarded tissue from surgical procedures following informed consent using IC-REC approved consent forms. Isolation of DP and PFI cells from biopsies was performed using a micro-dissection technique as previously reported [49]. Growth media (GM) consisted of DMEM supplemented with 10% FBS and 1% penicillin/streptomycin (P/S, Thermo Fisher, 15070-063). When osteogenic media (OM) was implemented, it consisted of low glucose DMEM (LG-DMEM, Thermo Fisher, 31885-023) containing 10% FBS and 1% P/S, and further supplemented with 100 nM dexamethasone (Sigma Aldrich, D4902), 50 μM l-ascorbic acid 2-phosphate (Sigma Aldrich, A8960) and 10 mM β-glycerol phosphate (Sigma Aldrich, G9422). Adipogenic media (AM) consisted of DMEM, 15% FBS, 1% P/S, 100 nM dexamethasone, 2.07 μM insulin (Sigma Aldrich, I9278), 0.5 mM 3-isobutyl-1-methylxanthine (IBMX, Sigma Aldrich, I7018), and 200 μM indomethacin (Sigma Aldrich, I7378). PFi were used from matched patient biopsies as the DP above. Rat DP were isolated from rat whiskers using the same technique [49], while human BM-MSC were purchased from Merck Millipore (SCC034).

### 2.3 Shock wave exposure

A compressed air-driven shock tube (length 4.13 m, internal diameter 59 mm) was used to propagate a shock wave over cells seeded in a 35 mm dish loaded onto an *ex vivo* organ culture (EVOC) rig located at the end of the shock tube as previously described [50]. Briefly, cells were seeded in 35 mm Petri dishes 24 h prior to shock wave exposure and incubated at standard culture conditions overnight. Following this, the media was removed and the dish was covered in a gas permeable membrane, and the cells were then exposed to a shock wave. Mylar diaphragms that varied in thickness were used to control the peak pressure of the shock wave. Post shock wave exposure the culture dish was refilled with fresh media appropriate for the test (GM, OM or AM). Cells could then be used immediately for testing or returned to standard culture conditions for short or long term assessment.

### 2.4 Assessment of cell viability and proliferation

Cell viability post shock wave exposure was assessed on human DP cells using both the Alamarblue assay (Thermo Fisher, DAL1100) and LIVE/DEAD imaging kit (Thermo Fisher, R37601). Three pressures were assessed, chosen to represent a low, medium and high pressure exposure, and were generated using Mylar diaphragms of thickness 0.023 mm, 0.050 mm and 0.125 mm, respectively. Positive and negative controls were included in the study: the positive control consisted of cells that were not exposed to a shock wave, while the negative control was treated with a 2% virkon solution for 30 s to initiate cell death. For Alamarblue, immediately following shock wave exposure, 2 ml GM was added to each dish along with 200 μl of Alamarblue blue reagent. Cells were incubated at standard culture conditions in the dark for 4 h, then absorbance was measured at 570 nm/600 nm using a plate reader. Media containing Alamarblue reagent was replaced with fresh GM and a second time point was taken at 24 h. Proliferation was assessed using the same Alamarblue method as previously described on human DP cells 1, 4 and 7 days post shock wave exposure. For LIVE/DEAD test samples, cells were incubated with the kit reagent and imaged 24 h post shock wave exposure using a fluorescence microscope. Quantitative analysis was performed by manually counting live and dead cells using ImageJ software (Version 1.49v).

### 2.5 Quantification of osteogenesis

Both a calcium assay and alizarin red were used as end point markers of osteogenesis to assess if exposure to a shock wave accelerated the rate of mineralisation. Four groups were evaluated: GM control, OM control, GM + shock wave (SW) and OM + SW. GM + SW and OM + SW were both exposed to a single 165 kPa shock wave and then were immediately refilled with either fresh GM or OM. Control groups were treated identically, except they were not exposed to a shock wave. The media was replaced with fresh media after 7 days in culture. When mineral deposits were observed, cells were immediately harvested for osteogenesis analysis. Time points of analysis are reported in the figure legends.

Calcium concentration was assessed using the Quantichrom calcium assay kit (Universal Biologicals, DICA-500) as per the manufacturer's instructions. Briefly, cells were washed twice in 1 ml of PBS then incubated at room temperature for 60 min on a rocking plate with 400 μl of 1 M hydrochloric acid solution (HCL, Fisher). Following this, a 20 μl aliquot from each dish was combined with assay reagent and the absorbance was measured at 612 nm using a plate reader.

Cells for alizarin red S staining were washed with PBS, then fixed using 4% paraformaldehyde (PFA) and stained using alizarin red solution (Merck, TMS-008-C). Images of mineral deposits stained with alizarin red were obtained at this point. Quantification was performed by incubating the samples in 800 μl of 10% acetic acid for 30 min on a rocking plate at room temperature. A 150 μl aliquot was taken from each sample and using a plate reader the absorbance was measured at 405 nm.

In addition to the osteogenic end point assay, a live cell capture system (Incucyte, Essenbioscience) was used to record the process of cell differentiation and mineral deposition. Human DP cells were set up and exposed to a shock wave as previously described, then loaded into an Incucyte microscope. Over a period of 12 days, an image of the entire Petri dish was taken every 2 h using a ×10 objective lens. These images were then formed into a video set to a speed of 3 frames per second.

### 2.6 Gene expression analysis

Reverse transcriptase quantitative polymerase chain reaction (RT-qPCR) was performed to identity any changes in osteogenic gene expression occurring in human DP cells between OM and OM + SW samples. Glyceraldehyde-3-phosphate dehydrogenase (GAPDH) was used as the housekeeping control. Shock wave exposed cells were harvested for RNA isolation. The cells were homogenized by centrifugation through Qiashredders (Qiagen, 79654), and RNA was isolated using a commercially available kit as per the manufacturer's instructions (Qiagen, RNeasy Plus Micro kit, 74034). A total of 100 ng of isolated RNA was then synthesised into cDNA by reverse transcription (SuperScript III Reverse Transcriptase, Thermo Fisher, 18080-093). To quantify mRNA expression, quantitative PCR was performed using a StepOnePlus system (Applied Biosystems). cDNA was combined together with H_2_O and Syber reagents (PowerUp Syber Green Master Mix, Thermo Fisher, A25779), with primers for RUNX2, DLX5 and GAPDH taken from Farshdousti Hagh et al. [51] (Table S1). RT-qPCR reactions were performed in quadruplicate. The thermocyclic conditions included an initial hold stage of 50 °C for 2 min then 95 °C for 2 min, followed by 40 cycles of 95 °C for 15 s and 60 °C for 1 min. Results were normalised to GAPDH and calculated using the ΔΔCt method.

### 2.7 Immunofluorescence and image analysis

Markers of cell morphology, histone modifications and nucleolin in human DP cells were imaged using fluorescence microscopy. For f-actin, vimentin and β-catenin, cells were fixed 24 h after shock wave exposure using 4% PFA. Blocking and permeabilisation of the cells was performed by incubation with a PBS solution containing 5% goat serum (Vectorlabs, S-1000) and 0.3% triton X-100 (Sigma Aldrich, X-100) for 30 min at room temperature (RT). After washes in PBS, cells were incubated for 1 h at RT with a primary antibody for either vimentin (Abcam, ab24525, 1:300) or β-catenin (BD Bioscience, 610153, 1:100) diluted in 5% goat serum in PBS. The cells were then washed in PBS and incubated for 1 h in the dark at RT with Alexa fluor 594 goat anti-chicken (Thermo Fisher, A11042, 1:200) diluted in PBS for vimentin, or Alexa fluor 488 goat anti-mouse (Thermo Fisher, A11001, 1:500) for β-catenin. After PBS washes, vimentin samples were counterstained with the f-actin marker Alexa fluor 488 phalloidin (Thermo Fisher, A12379, 2.5:100) in PBS for 30 min at RT in the dark. After final PBS washes, the coverslips were mounted on glass microscope slides using mounting media containing the nuclear stain DAPI (Vectorlabs, H-1200). Images were obtained using a fluorescence microscope fitted with a ×20 objective lens. For H3K4me3 and H3K27me3, cells were fixed 6 h after shock wave exposure, while cells to be stained for nucleolin were fixed after 24 h. All samples were fixed, blocked and permeabilised as previously described. Both primary and secondary incubation were as previously described, however antibodies were replaced with H3k27me3 (Abcam, ab6002, 1:200), H3k4me3 (Abcam, ab8580, 1:1000) and nucleolin (Abcam, ab136649, 1:200). Secondary antibodies included Alexa fluor 488 goat anti-mouse (Thermo Fisher, A11001, 1:500) and 594 goat anti-rabbit (Thermo Fisher, A11012, 1:500). Intensity of histone mark staining per nuclei was then calculated using ImageJ and normalised to the average intensity of DAPI. The number of nucleolin spots per nucleus was then counted manually using ImageJ (Version 1.49v).

To assess if the nuclear architecture of the human DP cells was perturbed by shock wave exposure, nuclear area was measured over a time course on samples in either GM, AM or OM. Cells were seeded in GM on sterile glass coverslips, incubated overnight in standard culture conditions and exposed to the shock wave. Immediately after shock wave exposure media was replaced with either GM, AM or OM. At 2, 6, 12 and 24 h post shock wave exposure, cells were fixed using 4% PFA. The cells were washed in PBS and then mounted on glass microscope slides using mounting media containing the nuclear stain DAPI. Images were obtained using a fluorescence microscope fitted with a ×20 objective lens. The nuclear area was then measured using ImageJ image analysis software (Version 1.49v).

### 2.8 Reduced representation bisulfite sequencing (RRBS)

Methylation profiling was performed to identify epigenetic modifications to DNA that may be occurring following shock wave exposure. Human DP cells were harvested 6 h post shock wave exposure. Genomic DNA (gDNA) was isolated using a commercially available kit (Zymo, Quick DNA Kit, D4068) following the manufacturer's recommended instructions. As a final step to increase the concentration of gDNA, concentrator columns (Zymo, Spin IC-XL, C1002-25) were used. 100 ng of gDNA per sample was sent to Diagenode for RRBS methylation profiling. To identify differentially methylated CpGs, comparative analysis on the RRBS datasets was performed with methylKit [52] using hg19, refGene and CpG island annotation from UCSC [53] (methylation difference cut-off of 25% and q-value of ≤0.01). Comparisons included GM *vs*. GM + SW, GM *vs*. OM, GM *vs*. OM + SW and lastly OM *vs*. OM + SW. Gene lists were generated where at least 1 differentially methylated CpG was located within the promoter region of that gene. Venn diagrams were then generated using Venny, and used to assess the distribution of hypo- and hyper-methylated gene promoters across the different conditions. SeqMonk was used to ascertain if differentially methylated CpGs located within gene promoters affected the entire 2 kb region, when comparing OM *vs*. OM + SW. We then assessed the influence of methylation on transcriptional activity by performing RT-qPCR as previously described, using mRNA isolated from DP cells at 6 and 24 h post shock wave exposure. Primers for RT-qPCR were designed for genes containing one or more differentially methylated CpGs within its promoter region and included ITGAV, MAP2K2, KCP, DEF6, DLK1 and CEBPB (Table S1). For our generated gene lists, hypomethylated promoters were given a pseudo +1 expression value while hypermethylated genes were labelled −1, then core analysis was performed using IPA (QIAGEN Inc., https://www.qiagenbioinformatics.com/products/ingenuity-pathway-analysis). Lastly, from the IPA core analyses, the top 5 physiological system development and functions for each condition was displayed as a bubble graph, with the size of the bubble representative of the number of genes associated with that category.

### 2.9 Bisulfite sequencing

To validation the RRBS data-set, MethPrimer [54] was used to design primers which covered more than one differentially methylated CpG within the specific areas of the promoter region of ITGAV, DLK1 and DEF6 identified in the RRBS analysis (Table S1), enabling amplification and bisulfite sequencing to be performed. Similar to the RRBS, build hg19 was used to obtain sequences for promotor regions of interest (ROI), locations for which are shown in the bisulfite sequencing figures. Briefly, gDNA from both human DP and BM-MSC, in conditions OM and OM + SW, were isolated as previously described in the section Reduced representation bisulfite sequencing. Bisulfite conversion was then performed on 50 ng of gDNA using the EZ DNA Methylation Gold kit (Zymo, D5005S) as per the manufacturer's instructions. Bisulfite PCR was performed using the following thermocyclic conditions; an initial hold stage of 95 °C for 2 min, then ×40 cycles of 95 °C for 20 s, 20 s at a primer dependent annealing temperature and 72 °C for 20 s. Following that the final stage was a hold stage at 72 °C for 5 min. Ligation of PCR products into a TOPO 4 vector (Thermo Fisher) was performed for 10 min at RT. Ligated products were then transformed into TOP10 cells by incubating on ice for 10 min, followed by heat shock at 42 °C for 30 s. SOC medium was then added to the TOP10/ligation mix and cells were incubated at 37 °C on a shaker for 1 h, prior to being spread on LB-agar plates containing 50 μg/ml ampicillin and 40 μg/ml X-gal. Plates were incubated for 14 h at 37 °C. White colonies containing the PCR-product were then transferred individually into LB broth with ampicillin and incubated at 37 °C on a shaking incubator for 14 h. 150 μl of broth containing the sample was directly sequenced (Source Bioscience, Bugs2bases service). Sequences were read using Codon Code Aligner 8.0.2, enabling the methylation status of individual CpGs to be assessed.

### 2.10 Drug treatment: cilengitide

To test if we could abrogate the effect of the accelerated mineralisation we observed in our cell culture model, we purchased the ITGAV/B3 and ITGAV/B5 integrin inhibitor molecule cilengitide from Sigma Aldrich (SML1594). Using a concentration of 10 μM, that has been previously reported to enhance adipogenesis in the presence of AM without significantly comprising cell proliferation [55], cilengitide was added to the OM of shock wave exposed DP and BM-MSCs, as well as the GM of cells not exposed to the shock wave. As a positive control cells were also cultured in OM and GM without the presence of cilengitide. After 14 days in culture, staining for the presence of mineral deposits was performed using Alizarin Red S which is described in detail in the Quantification of osteogenesis section. Viability of BM-MSCs cultured in OM with the addition of 10 μM, or a range of concentrations, including 1 nM, 10 nM, 100 nM, 1 μM and 10 μM were assessed using the previously described method in section Assessment of cell viability and proliferation. Lastly, after 21 days in culture, using the same concentrations of cilengitide as in the viability test, BM-MSCs were assessed for mineral deposition using the same method as described in Quantification of osteogenesis.

### 2.11 Statistics

Statistical analyses were performed in GraphPad Prism (Version 6). Statistical differences among conditions were identified using the one-way, and when appropriate, the two-way analysis of variance test (ANOVA), combined with either the Tukey's or Dunnett's multiple comparisons test. When two conditions were present, statistical differences were assessed using a two tailed Student's *t*-test. A p value of ≤0.05 was deemed to be statistically significant.

### 2.12 Data availability

All relevant data are available from the authors upon request.

## 3 Results

### 3.1 A single shock wave can accelerate osteogenic differentiation

**Fig. 1:**
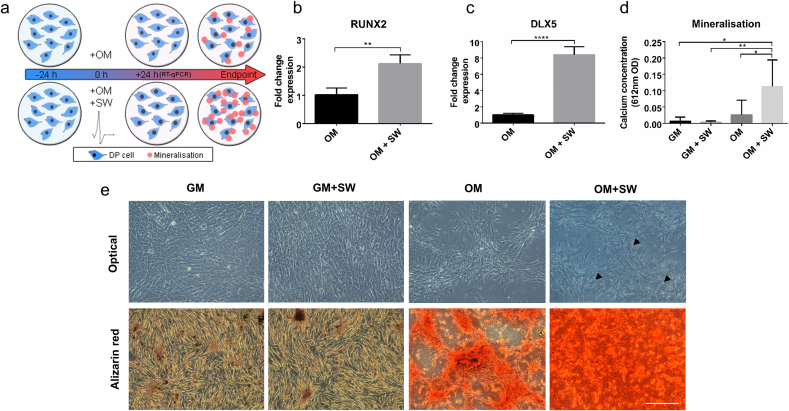
Osteogenic differentiation increases in cells exposed to a SW in OM. (a) Illustration depicting the experimental workflow used to assess changes in mineralisation from shock wave exposure. (b and c) RT-qPCR analysis of Runx2 and Dlx5 showing a significant increase in the expression of these osteogenic associated genes in OM + SW relative to OM at 24 h in human DP cells. Each bar represents the mean ± 1 SD, n = 4. ** = p ≤ 0.01, **** = p ≤ 0.0001, Student's *t*-test (d) Assay showing late stage mineralisation in human DP cells through the amount of calcium deposition 8 days post shock wave exposure. Significantly more calcium was detected in OM + SW compared to the other groups. Each bar represents the mean ± 1 SD, n = 5. * = p ≤ 0.05, ** = p ≤ 0.01, one way ANOVA plus Tukey's multiple comparisons test. (e) Representative optical images showing the early deposition of bright mineral nodules on OM + SW (black arrow heads) in human DP cells. Alizarin red staining of rat DP cells showing same effect as observed in human DP. Red staining shows the presence of mineral which is more abundant in OM + SW compared to all other conditions. Scale bar = 300 μm.

As we were trying to model trauma induced HO, we first wanted to identify the maximum shock wave pressure that could be used without inadvertently causing cell death. When we assessed cell viability we found that cells remained viable when exposed to shock waves with a peak pressure up to 165 kPa (Fig. S1). At pressures higher than this, cells would rip off the plate in sheets. We therefore chose to expose cells to a maximum shock wave of 165 kPa in subsequent experiments. Before assessing osteogenic differentiation by quantifying mineralisation, we looked to see whether early changes were induced by exposure of human DP cells to a shock wave, such as variances in expression of osteogenic master regulators (Fig. 1a). We found that Runt-related transcription factor 2 (RUNX2) expression was significantly increased (Fig. 1b) by 2 fold in osteogenic media + shock wave (OM + SW, Mean ± SD 2.127 ± 0.307) when compared to osteogenic media (OM, 1.025 ± 0.238) alone. The RUNX2 activator, distal-less homeobox 5 (DLX5) was also found to have increased 8 fold (Fig. 1c) in OM + SW (8.398 ± 0.993) when compared to the OM control (1.013 ± 0.180). No changes were observed in cells in growth media (GM) in response to a shock wave alone (data not shown). However, considering that these osteogenic master regulators were significantly up regulated just 24 h after exposure of cells to a shock wave in OM, we decided to determine whether the shock wave also promoted an increase in osteogenic differentiation, and notably end point mineralisation, in either OM or GM. A live cell imaging microscope (Incucyte) in combination with a calcium assay were used to observe the onset and determine the amount of mineralisation occurring in all conditions over several days. We found that calcium was deposited more rapidly (Movie S1) and at significantly higher amounts in cells cultured in OM + SW (Fig. 1d, Mean ± SD, OM + SW = 0.112 ± 0.081) when compared to all other conditions (Mean ± SD, GM = 0.007 ± 0.012, GM + SW = 0.003 ± 0.004, OM = 0.026 ± 0.044). It is important to note that the shock wave did not accelerate proliferation of cells compared to controls, (Fig. S1e) and so the described effects were not due to more cells being present. Next we used rodent DP cells and assessed both calcium ion content (Fig. S2a) and total mineralisation with alizarin red staining (Fig. 1e). We found the same osteogenic effect was observed in rodent DP as in human DP, with strikingly more staining for mineral present in cells exposed to OM + SW compared to all other conditions. Overall these results suggest there is a synergistic effect of the OM together with exposure to a shock wave that results in acceleration of osteogenic differentiation and mineralisation in both human and rodent DP cells. We also assessed the effect of OM + SW on human BM-MSCs and found more mineral was present in the OM + SW condition compared to OM (Fig. S2b). To determine if this was a shock wave specific response we assessed the effect of the shock wave on another human cell type, dermal PFi, the aforementioned sister cell type of DP cells. We found that neither OM nor OM + SW had any effect on mineralisation in dermal PFi (Fig. S2c) implying that the effect of the shock wave is not transferable to all mesenchymal cell types. Considering the high prevalence of inappropriate ossification in blast causalities suffering with HO, the increased osteogenic capacity of the shock wave exposed DP cells in our model, combined with the lack of ossification in a biologically matched control cell type, we conclude that human DP are an ideal model cell type to determine the epigenetic basis of inappropriate ossification, otherwise known as trauma-induced HO.

### 3.2 Shock wave-induced OM accelerates changes to nuclear architecture

**Fig. 2:**
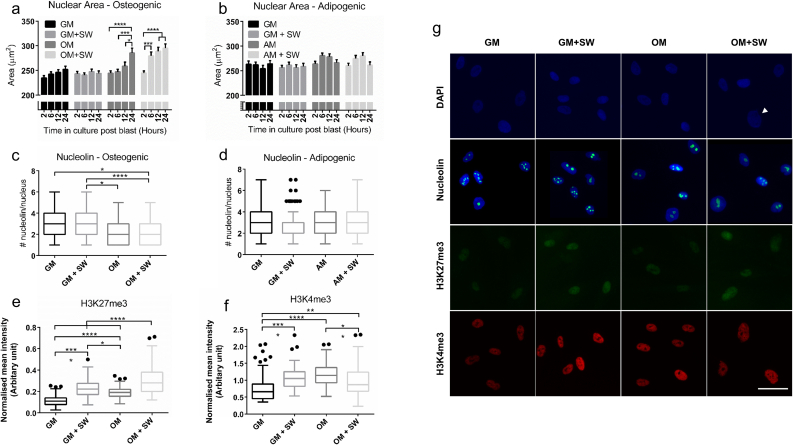
SW exposure in the presence of OM leads to changes in nuclear architecture in human DP cells. (a) Nuclear area of cells cultured in OM. Cells in OM + SW show an expansion in nuclear area after 6 h, which was not observed until 24 h in the OM. No change to nuclear area was observed in GM samples. Each bar represents the mean ± standard error of the mean (SEM), n = 105. * = p ≤ 0.05, *** = p ≤ 0.001, **** = p ≤ 0.0001, two way ANOVA. (b) Nuclear area of cells in AM. The nuclear area of cells in GM or AM was not affected. Each bar represents the mean ± 1 SEM, n = 146, two way ANOVA. (c) Nucleolin spots within cell nuclei 24 h post shock wave exposure in OM. There was no effect of the shock wave although significantly fewer nucleolin spots were observed in cells cultured in OM compared to GM. For both c and d, each box plot represents the upper and lower quartiles with Tukey whiskers, n = 100. * = p ≤ 0.05, **** = p ≤ 0.0001, one way ANOVA. (d) Nucleolin spots within cell nuclei 24 h post shock wave exposure in AM. No significant changes were observed. (e) H3K27me3 intensity quantification at 6 h. OM + SW cells had the strongest expression of H3K27me3, followed by GM + SW, OM and lastly GM. For e and f, each box represents the upper and lower quartiles with Tukey whiskers, n = 80. * = p ≤ 0.05, ** = p ≤ 0.01, **** = p ≤ 0.0001, one way ANOVA. (f) H3K4me3 intensity quantification at 6 h. A greater H3K4me3 signal was observed in GM + SW cells compared to GM. This effect was not present in OM. (g) Representative images showing increased nuclear area in OM + SW cells (white arrow) using blue DAPI stain. Nucleolin spots are shown as green counterstained with DAPI in blue. H3K27me3 is shown as green, while H3K4me3 is shown as red. Scale bar = 50 μm. All ANOVA tests were followed by a Tukey's multiple comparisons test. (For interpretation of the references to colour in this figure legend, the reader is referred to the web version of this article.)

After identifying that shock waves together with OM can accelerate osteogenic differentiation compared to OM alone, we postulated that there would be nuclear changes induced early on by the shock wave. As the nuclear volume of MSCs has been shown to change during osteogenic differentiation and is affected by mechanical stress [56], we decided to assess the architectural structure of cell nuclei to identify any changes over time that may be occurring due to either the shock wave or culture media. In OM + SW, a significant enlargement in nuclear area was observed after only 6 h post shock wave exposure (Fig. 2a) (Mean ± SEM, OM + SW: 2 h = 244.7 ± 4.1, 6 h = 279.4 ± 7.8, 12 h = 288.8 ± 8.2, 24 h = 295.2 ± 8.4) which was not present in any other condition at that time point. Surprisingly, no significant changes in nuclear area were observed over the entire time course in either the GM or GM + SW conditions (Mean ± SEM, GM: 2 h = 234.5 ± 4.8, 6 h = 242.4 ± 4.8, 12 h = 245.9 ± 5.6, 24 h = 252.1 ± 6.2. GM + SW: 2 h = 243.2 ± 4.8, 6 h = 240.4 ± 4.5, 12 h = 247.5 ± 5.0, 24 h = 243.8 ± 5.2) indicating that the shock wave alone had no effect on cells. However, 24 h post the start of the experiment a significant increase in nuclear area was observed in the OM condition (Mean ± SEM, OM: 2 h = 244.5 ± 4.7, 6 h = 247.0 ± 5.2, 12 h = 258.5 ± 8.3, 24 h = 285.4 ± 9.7) suggesting a delayed response compared to the OM + SW condition. This again highlights that the cells respond to both the shock wave and OM in a synergistic manner. To assess if this accelerated enlargement in nuclear area also occurred in other differentiation conditions, an identical experiment was performed using adipogenic media (AM) in place of OM and no significant changes to nuclear area were observed (Fig. 2b, Mean ± SEM, GM: 2 h 263.3 ± 6.2, 6 h = 262.0 ± 5.4, 12 h = 254.3 ± 6.7, 24 h = 263.9 ± 6.7. GM + SW: 2 h = 256.2 ± 4.7, 6 h = 261.6 ± 5.6, 12 h = 256.0 ± 6.1, 24 h = 258.6 ± 6.3. AM: 2 h = 263.9 ± 5.1, 6 h = 280.8 ± 5.1, 12 h = 278.5 ± 5.7, 24 h = 266.4 ± 5.9. AM + SW: 2 h = 260.0 ± 5.3, 6 h = 274.9 ± 6.1, 12 h = 280.1 ± 6.5, 24 h = 261.1 ± 6.8), suggesting the accelerated nuclear size expansion occurring as a result of shock wave exposure is osteogenic specific.

We next attempted to identify whether the shock wave-induced changes in nuclear morphology led to other nuclear re-modelling. We assessed the distribution of the nuclear protein nucleolin, which is known to act as a histone chaperone [57], and found that while there did not appear to be any effect of the shock wave (Fig. 2c), there was a significant drop in the number of nucleolin spots per nuclei in OM and OM + SW when compared to GM and GM + SW at 24 h (Mean ± SD: GM = 2.74 ± 1.4, GM + SW = 2.99 ± 1.4, OM = 2.49 ± 1.2, OM + SW = 2.22 ± 1.1). Again, to test if the change in nucleolin was osteo specific, we repeated the experiment in AM and found no significant changes in the number of nucleolin per nuclei (Fig. 2d, Mean ± SD: GM = 2.82 ± 1.3, GM + SW = 2.81 ± 1.4, AM = 2.9 ± 1.2, AM+SW = 3.0 ± 1.4).

As were able to see a change in nucleolin number in cells cultured in osteogenic conditions compared to GM, but no effect of the shock wave, we decided to assess specific markers of histone modifications commonly associated with either heterochromatin or euchromatin. We found that H3k27me3, a widely recognised marker of heterochromatin, had the highest intensity in cells in the OM + SW condition (Fig. 2e). Both GM + SW and OM also had a significant increase in H3K27me3 expression compared to GM (Mean ± SD: GM = 0.11 ± 0.05, GM + SW = 0.23 ± 0.08, OM = 0.19 ± 0.05, OM + SW = 0.30 ± 0.14). The expression of euchromatin marker H3K4me3 did not follow any trend observed in H3K27me3 (Fig. 2f), although significant changes between the conditions were again observed, with the greatest expression observed in GM + SW and OM (Mean ± SD: GM = 0.77 ± 0.41, GM + SW = 1.08 ± 0.33, OM = 1.16 ± 0.32, OM + SW = 0.96 ± 0.45).

As well as nuclear changes we also assessed morphological changes within the cell body by fluorescently labelling a cytoskeletal marker, filamentous actin (f-actin), and staining for the mesenchymal intermediate filament protein vimentin at 24 h. Cells in OM and OM + SW conditions appeared larger and more spread compared to those cultured in GM conditions (Fig. S3). In addition, the expression of vimentin appeared to be reduced within cells in the OM conditions, signifying that changes to the cell structure are occurring in both OM and OM + SW conditions compared to GM (Fig. S3).

Together, the observations that include increased nuclear area, histone chaperone distribution and histone mark expression, suggest that shock wave-induced changes to nuclear organisation are occurring in OM. The accelerated nuclear expansion in the OM + SW condition, combined with their unique histone modification expression at 6 h indicate that human DP cells at this time point may be in the process of reorganising their nuclear structure into a configuration that could have the potential to accelerate differentiation along their osteogenic lineage.

### 3.3 A single shock wave alters DNA methylation profiles

**Fig. 3:**
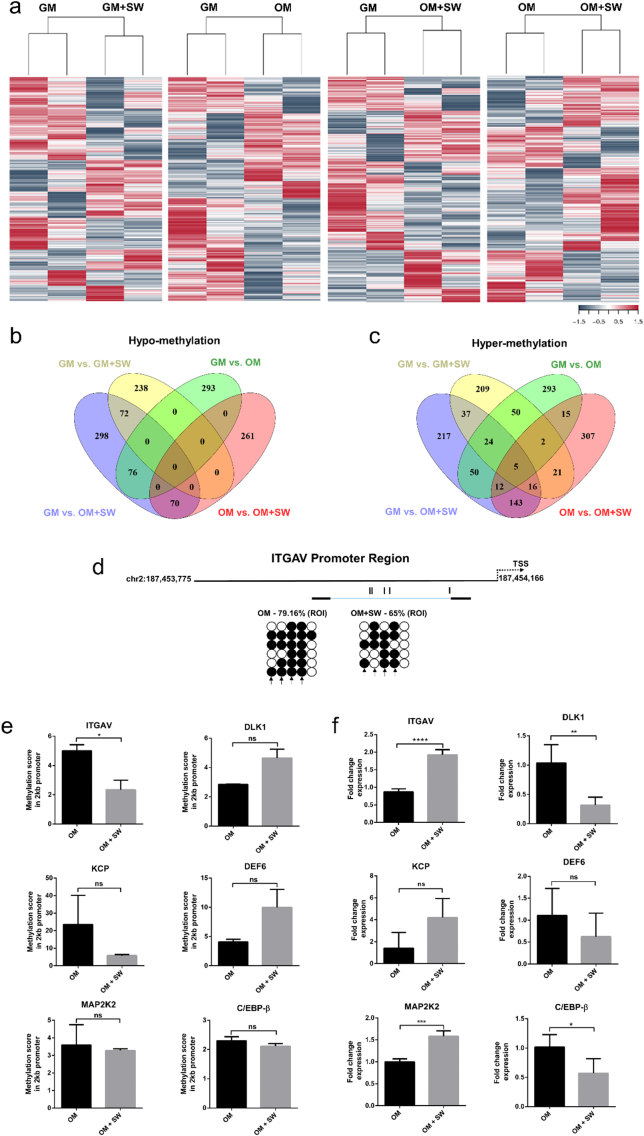
Application of a SW can alter DNA methylation in gene promoters leading to opposing changes in gene expression. (a) Heat maps showing differentially methylated regions between GM *vs*. GM + SW, GM *vs*. OM, GM *vs*. OM + SW and OM *vs*. OM + SW generated through reduced representation bisulfite sequencing of two biological replicates of human DP cells. (b) Venn diagram of hypo-methylated genes in each condition. (c) Venn diagram of hyper-methylated genes in each condition. (d) Bisulfite sequencing of a region of interest (ROI) located in the ITGAV promotor in human DP cells in OM and OM + SW conditions (filled circles represent a methylated CpG site while open circles represent no methylation) shows hypo-methylation of the promotor in response to the shock wave. Arrows indicate CpGs that fall within the ROI identified in RRBS analysis (e) Methylation score profiles of 2 kb promoter region found upstream of transcription start site generated using SeqMonk. Each bar represents the mean ± 1 SD. N = 2. * = p ≤ 0.05, ns = not significant, Student's *t*-test. (f) RT-qPCR validation of hypo and hyper-methylated genes in human DP cells that show methylation scores were inversely correlated with changes in gene expression. Hypo-methylated genes include ITGAV, MAP2K2 and KCP. Hyper-methylated genes include DLK1, DEF6 and C/EBP-β. Each bar represents the mean ± 1 SD. N = 4. ** = p ≤ 0.01, **** = p ≤ 0.0001, Student's *t*-test.

Given the organisational changes in cell nuclei in OM + SW conditions at 6 h (Fig. 2), we decided to investigate the epigenetic machinery that may be enabling our observed acceleration of osteogenic differentiation and mineralisation in the OM + SW condition (Fig. 1), at this same time point. As approximately 70% of gene promotors contain CpG islands [58], whose activity is governed by their methylation status [59], we performed reduced representation bisulfite sequencing (RRBS) on gDNA isolated from human DP cells at the 6 h time point, with the aim of identifying CpGs located within gene promoters which are differentially methylated as a result of shock wave exposure or change into osteogenic media (Figs. S4 and S5, Table S2). To identify differentially methylated regions, comparative analysis was performed on GM *vs*. GM + SW, GM *vs*. OM, GM *vs*. OM + SW and OM *vs*. OM + SW. Performing hierarchical clustering revealed similarity between biological replicates, yet differences between conditions (Fig. 3a). From the comparative analyses we generated gene lists for both hypo and hyper-methylated genes, using the inclusion criteria that at least 1 differentially methylated CpG was located within the promoter region of that gene (Table S3). As our main interest was trying to identify what may be causing the acceleration in osteogenic differentiation seen in OM + SW cells, we used Venn diagrams to narrow our focus on differentially methylated gene promoters that were exclusive to the OM + SW condition when compared to either GM or OM (Fig. 3b,c). We further narrowed our focus by investigating genes which showed >1 differentially methylated CpG in their promotor. Hypo-methylated genes included the mechanosensor ITGAV and bone morphogenetic protein (BMP) signalling enhancer Kielin/Chordin-Like Protein (KCP) [60]. ITGAV, which is found in focal adhesions, is known to increase in expression in BM-MSCs undergoing osteogenic differentiation [61], while gene knockdown in adipose stem cells can promote adipogenic differentiation [55]. KCP has also been found to increase BMP signalling in a paracrine manner, where KCP binds to BMP7 and enhances binding to the type I receptor [60]. Hyper-methylated genes included the adipose progenitor cell marker Delta Like Non-Canonical Notch Ligand 1 (DLK1) [62] and DEF6 Guanine Nucleotide Exchange Factor (DEF6), thus it seems there may be a dichotomy between osteogenic enhancement and adipogenic inhibition in terms of hypo- and hyper-methylated promotors. To validate our RRBS findings we then performed bisulfite sequencing on new gDNA samples isolated from both human DP cells and BM-MSC, in OM and OM + SW conditions at the same 6 h time point (Figs. 3d, S6). We found that hyper-methylation and hypo-methylation of regions of interest within gene promoters identified in RRBS analysis was again present with trends following the correct direction in both DP cells and BM-MSC, however, the impact of the shock wave on altering methylation was not always as striking as that identified in the RRBS. As the bisulfite sequencing validation was performed on newly generated gDNA samples from primary human cells, minor differences between how the cells reacted to the shock wave will have been present and perhaps the cells may have required additional time post shock wave exposure for larger changes to methylation to occur fully. To investigate the RRBS methylation data further, using the bioinformatics tool, SeqMonk, we were able to create promoter regions 2 kb upstream of the transcription start site and ascertain an overall methylation score of that region of interest. Genes where multiple differentially methylated CpGs were identified within the promoter showed clear changes in methylation score in OM *vs*. OM + SW conditions (Fig. 3e, Mean ± 1 SD. ITGAV; OM = 5.01 ± 0.42, OM + SW = 2.35 ± 0.65. KCP; OM = 23.53 ± 16.64, OM + SW = 5.8 ± 0.58. DLK1; OM = 2.85 ± 0.01, OM + SW = 4.64 ± 0.62. DEF6; OM = 4.07 ± 0.47, OM + SW = 9.99 ± 3.10). Of our genes of interest, we also included one hypo (the MAPK/ERK activator Mitogen Activated Protein Kinase Kinase MAP2K2) and one hyper-methylated (the adipogenesis regulator CCAAT/Enhancer Binding Protein Beta C/EBP-β [63]) gene where only a single differentially methylated CpG was located within its promoter region. Despite these genes having been identified in our original comparative analysis, with SeqMonk we found that the overall methylation score of the promoter region for these genes was not significantly altered (Fig. 3e, Mean ± 1 SD. MAP2K2; OM = 3.60 ± 1.16, OM + SW = 3.28 ± 0.10. C/EBP-β; OM = 2.31 ± 0.13, OM + SW = 2.11 ± 0.09).

We next wanted to assess the robustness of our data and determine if changes to differential methylation could result in altered transcriptional activity. We performed RT-qPCR and were able to identify trends between hypo-methylated genes resulting in increased transcription (Fig. 3f, Mean ± 1 SD. ITGAV; OM = 0.87 ± 0.09, OM + SW = 1.93 ± 0.15. MAP2K2; OM = 1.01 ± 0.12, OM + SW = 1.34 ± 0.07. KCP; OM = 0.88 ± 0.7, OM + SW = 2.31 ± 0.84) and reduced expression in genes where hyper-methylation at the promoter was observed (Fig. 3f, Mean ± 1 SD. C/EBP-β; OM = 1.02 ± 0.21, OM + SW = 0.57 ± 0.25. DLK1; OM = 1.04 ± 0.31, OM + SW = 0.32 ± 0.14. DEF6; OM = 1.11 ± 0.61, OM + SW = 0.62 ± 0.54). What was especially intriguing was that for both MAP2K2 and C/EBP-β, where there was no overall change in the methylation score of the promoter region (Fig. 3e), transcriptional changes did still occur (Fig. 3f), implying that even in cases where only a single CpG is differentially methylated, this may be sufficient to influence transcriptional changes. These data demonstrate that our methylation profile datasets accurately represent differentially methylated CpGs within promoters of human DP cells, which lead to alterations in transcriptional activity.

### 3.4 Inhibition of ITGAV can abrogate mineralisation

**Fig. 4:**
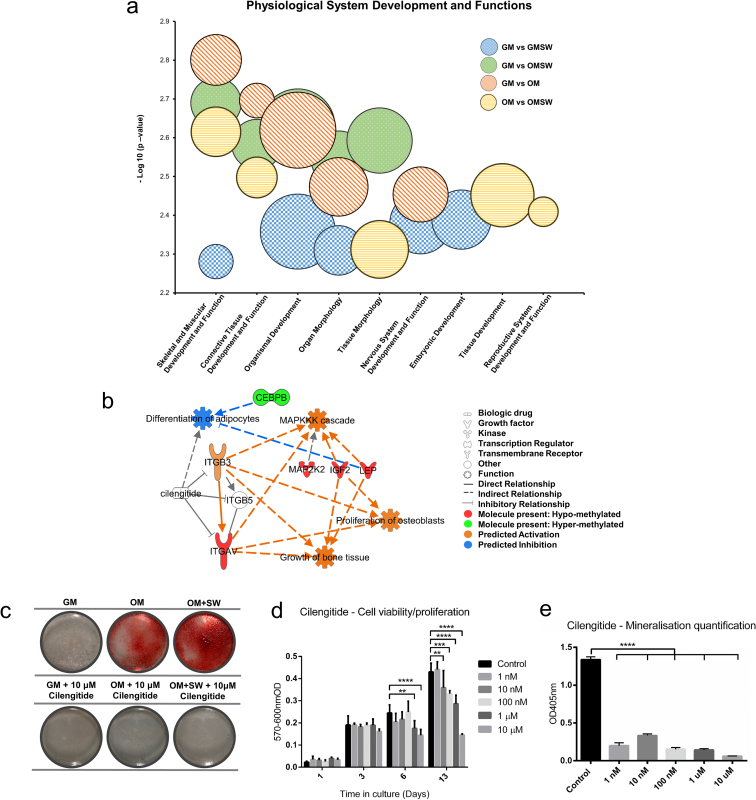
Targeting ITGAV using the drug cilengitide can lead to abrogation of mineral deposition (a) Visualisation of core analysis data generated in IPA from human DP RRBS data-sets, showing the top 5 physiological system development and function categories for each condition ranked through enrichment score using Fisher's exact test p-value. The size of each bubble represents the number of genes that are present within a category. p-values shown were generated within IPA using the Fisher's exact test and represent the mean of the sub categories present in each individual condition. (b) Interaction network for transmembrane receptor ITGAV in the OM *vs*. OM + SW condition generated using IPA knowledge base, showing the relationships with cilengitide, an ITGAV inhibitor. Molecules of interest and their influence on the activation or inhibition of adipogenic and osteogenic differentiation functions are shown. (c) Alizarin red staining showing representative whole well images of mineralisation in human DP cells after 14 days in culture in GM, OM, GM + 10 μM cilengitide and OM + 10 μM cilengitide in 35 mm Petri dishes. The presence of 10 μM cilengitide in OM neutralised the osteogenic induced differentiation potential of the OM and shock wave, blocking mineral deposition. Red staining indicates the presence of mineral deposition. (d) Drug concentration viability/proliferation time course showing that higher concentrations of cilengitide reduce the proliferative capacity of BM-MSCs over time. Lower concentrations of cilengitide appear to not affect proliferation/viability. Each bar represents the mean ± 1 SD. n = 4, ** = p ≤ 0.01, *** = p ≤ 0.001, **** = p ≤ 0.0001, two-way ANOVA, followed by Dunnett's multiple comparison test. (e) Alizarin red staining quantification data in human BM-MSCs after 21 days in culture showing that all tested cilengitide concentrations reduce mineral deposition. Each bar represents the mean ± 1 SD. n = 6, **** = p ≤ 0.0001, one way ANOVA. (For interpretation of the references to colour in this figure legend, the reader is referred to the web version of this article.)

Following our demonstration that changes in methylation status induced by a high-energy shock wave resulted in altered transcriptional activity, we performed core analyses on our gene lists using the Ingenuity Knowledge Base within Ingenuity Pathway Analysis software (IPA, Qiagen) [64] to ascertain biological meaning from the datasets. Visualisation of the top five physiological system development and functions for each condition using a bubble graph, showed clustering of the OM and OM + SW groups compared to GM *vs*. GM + SW in functions representing Skeletal and Muscular, and Connective Tissue Development (Fig. 4a). Intriguingly, Tissue Development was identified as one of the top 5 functions uniquely in the OM *vs*. OM + SW condition. When we explored this further by looking at the top 10 subcategory functions of that group, we found an enrichment of bone formation related annotations (Fig. S7a). Further exploration of the core analyses, specifically for Tissue Development, did uncover a high number of bone related annotations in both the GM *vs*. OM and GM *vs*. OM + SW subcategories, although these were not identified as the top physiological functions in the core analysis dataset, implying that within the OM *vs*. OM + SW gene list, there may be an enrichment towards the development and formation of bone tissue.

The Tissue Development bone annotations present in OM + SW condition, contained a number of genes with hyper and hypo-methylated promoters, including the aforementioned ITGAV. Using the IPA knowledge base we were able to perform an additional analysis in the form of a prediction network centred on ITGAV, to investigate its potential influence on other HO associated hypo- and hyper-methylated promoters (Fig. 4b). A number of the top functions affected by the generated network had osteogenic associated applications, including the growth of bone tissue and proliferation of osteoblasts. Interestingly, adipogenesis was predicted to be inhibited, implying that ITGAV activity may be involved in modulating adipogenic or osteogenic fate decisions. Increased expression of ITGAV is associated with osteogenic differentiation, while inhibition through gene knockdown promotes adipogenesis. Cilengitide is small molecule cyclopeptide, which inhibits binding and activation of both ITGAV/B3 and ITGAV/B5 [48], and use of this in cultures of adipose stem cells has previously been shown to elicit a similar effect to ITGAV gene knockdown, namely increased adipogenesis [55]. We therefore postulated that cilengitide might also serve to inhibit shock wave-induced osteogenesis. On testing this, we found that not only did cilengitide abrogate the accelerated differentiation effect of the shock wave, but it was able to completely prevent the onset of mineral deposition in human DP cells in OM (Figs. 4c, S7b). To determine if our cell culture model could accurately predict cell response in other mesenchymal cell types, we also wanted to test cilengitide on other cell types which can differentiate into bone. We found that on human BM-MSCs, treatment with cilengitide was again able to significantly abrogate osteogenesis (Fig. S7c,d), showing us the adaptability of our culture model system and supporting our position that DP cells are an appropriate cell type to effectively model HO. Clear morphological differences were present in BM-MSCs cultured in the presence of 10 μM cilengitide (Fig. S7d). To determine if cilengitide was reducing the mineralisation capacity of cells by having an inhibitory effect on pathways, or by decreasing cell viability, we performed a concentration viability/proliferation test to identify a dose that did not have a negative impact on cells. We found that a dose of 1 nM of cilengitide did not affect viability or proliferation (Fig. 4d) and when we tested this concentration on mineralisation, found that it was still effective in abrogating the onset of mineral in BM-MSCs after 21 days in culture. This data highlights that our cell culture model of trauma-induced HO can be used to identify therapeutics to inhibit inappropriate ossification that arise specifically from the mechanical perturbation of the shock wave *in vitro*, with the next step being to evaluate if our findings can be translated to prevent HO in an animal model *in vivo*, where a systemic response will also be present.

## 4 Discussion

With the aim of further understanding trauma-induced HO, we describe in this manuscript the development of a simple cell culture model system which enables assessment of cellular response to one component of the blast, specifically a 165 kPa shock wave in air. Using human mesenchymal DP cells to model inappropriate ossification *in vitro*, we show that a single shock wave can increase expression of osteogenic master regulators RUNX2 and DLX5, which ultimately lead to accelerated mineral deposition by cells (Fig. 1). To our knowledge, no other *in vitro* trauma-induced HO osteogenic models that use a compressed air-driven shock tube have been reported, however, animal HO models show similarities in the form of ectopic bone formation following blast-induced injury [[23], [24], [25]]. Using a cell culture model enables us to separate out the traumatic events caused by exposure to a blast wave, from the direct effect of the shock wave itself.

As we wanted to model trauma-induced HO, we chose to evaluate cellular response to a single high energy blast in air. Mechanical loading and osteogenesis is not new and others have reported enhanced osteogenic capacity *in vitro* using thousands of low energy ESWs over a period of time. In human BM-MSCs, ESW can promote enhanced osteogenesis by Ras activation [45], and cellular Adenosine Triphosphate (ATP) activation of P2X7 receptors [46]. Interestingly, application of ESW has been found to promote the opposite effect on adipogenic activity by inhibiting the process in human BM-MSCs [65]. Given their osteogenic differentiation capacity, ESW are used therapeutically in clinics to treat musculoskeletal conditions, where their low intensity pulses mean they do not inadvertently cause HO. While the ESW models discussed are significantly different to the trauma associated model reported in this study, they support the hypothesis that application of mechanical loading can transform cell identity into an enhanced osteogenic state.

As our model was able to transition cells *in vitro* into an enhanced osteogenic state, we wanted to explore the possible epigenetic machinery behind this. We identified 6 h as a post-blast time point of interest, as there was an increase in cell nuclear area at this time in the OM + SW condition, 18 h ahead of the observed increase in the OM control (Fig. 2a). This suggests that changes to nuclear architecture are occurring at an accelerated rate in cells in OM + SW. Of the many epigenetic mechanisms, we decided to focus our attention on DNA methylation. The methylation profile within the promoter region has been reported to play a regulatory role that affects the transcription of osteogenic genes, such as osteocalcin (OCN), DLX5, osterix and alkaline phosphatase (ALP) [51,66,67,68]. In our model using RRBS, we were able to identify hundreds of genes that contained at least a single differentially methylated CpG within their promoter region (Fig. 3b,c) following mechanical stimulation in the form of a shock wave. When multiple CpGs in a single promoter were detected as differentially methylated, we were able to see clear changes in the methylation score of the entire promoter region, but change within a single CpG was not sufficient to change the overall score of the promoter region (Fig. 3e). Intriguingly, in promoters that contained only a single differentially methylated CpG, we still identified changes in gene transcription (Fig. 3f), implying that a single differentially methylated CpG may potentially influence transcriptional changes, although other factors that influence transcription may also play a role. In a previous study that looked at the regulatory landscape of osteogenic differentiation in immortalised human BM-MSCs over 28 days in osteogenic culture, altered methylation status was found at 1273 CpG sites: 1121 sites with decreased methylation and 152 sites with increased methylation [69]. These changes in methylation status affected 710 genes, which when averaged, identified a total of 1.6 CpG sites per gene. This study concluded that DNA methylation remained relatively constant and was not important for governing the osteogenic differentiation of BM-MSCs. However, because we are able to see transcriptional changes that may result from a single differentially methylated CpG, our data suggest that DNA methylation may play a far more important role in the osteogenic differentiation of mesenchymal cells than previously thought.

Application of mechanical strain on rat BM-MSCs has been reported to impede adipogenic differentiation and enhance osteogenic differentiation [70]. Mechanical cues that affect DNA methylation have also been reported, such as the application of dynamic fluid flow on murine BM-MSCs causing a decrease in the methylation of the osteopontin (OPN) promoter, which ultimately led to increased OPN transcription [71].

IPA analyses on our methylation data identified OM *vs*. OM + SW as containing a number of skeletal and osteogenic development associated genes which were hypo-methylated, alongside several hyper-methylated genes which were associated with adipogenesis. When we identified that there was a drug, cilengitide, which inhibited one of our hypo-methylated genes, the transmembrane receptor integrin ITGAV, and could also accelerate adipogenesis, we postulated that it acted by shifting the delicate balance between osteo- and adipo-genic differentiation towards an adipogenic fate [47]. When we tested this drug as a means to abrogate shock wave-induced osteogenesis, we found that on DP cells in OM, or OM + SW, we completely averted mineral deposition (Figs. 4c and S7b). Further testing of cilengitide on human BM-MSCs showed similar results to in DP cells (Fig. S7c,d), highlighting the adaptability of our cell culture model. Thus, as a preventative treatment for HO, cilengitide may serve to inhibit sensitisation of cells to an osteogenic identity, and in turn prevent ectopic bone formation.

In this study we focused on analysing the cellular response to a shock wave, as a single component of blast, and identified the integrin ITGAV as a molecule of interest for its potential role in the production of ectopic mineral deposition. Interestingly, the recent identification of potential factors that may play a role in HO, which are elevated in the serum of patients exposed to blast, show some similarities with our shock wave *in vitro* model. Proteomic biomarker analysis on human serum from healthy and HO subjects combined revealed enrichment of B1 and B3 integrin cell surface interactions. Further network analysis on all serum proteins that were differentially expressed between subjects with and without HO, displayed pathway enrichment for extracellular matrix–receptor interactions [72], inclusive of integrins. In a separate study, serum from civilian and military subjects with trauma-induced HO was found to alter the gene profile of human adipose derived stem cells (ASCs), by impacting the MAPK signalling pathway [73]. Similarities in the altered regulatory pathways between serum HO and blast-*in vitro* HO models suggest they may play a similar role in the initiation of an osteogenic cell phenotype, however, one outstanding question in the serum HO model is with regard to which cells secrete factors leading to the systemic response seen in response to blast. Understanding this would help us to elucidate if HO is caused by a cell autonomous, or non-cell autonomous effect. Despite this, blast-induced HO is still a complex disease that is influenced by multiple factors such as the initial injury obtained by blast, and the systemic response to trauma. Considering the similarities in HO serum models [72,73] and the present blast-model, the next step would be to test the efficacy of the ITGAV inhibitor cilengitide in a blast-HO animal model, where all the factors from blast-induced trauma will be present.

## 5 Conclusions

In summary, we have shown here that a single high energy shock wave can accelerate osteogenic differentiation in DP and BM-MSCs, but not in a DP sister cell type known as PFi. We also revealed that a shock wave can induce changes in the methylation status of gene promoters, leading to altered transcription profiles. Finally, focusing our attention on ITGAV, whose promoter is hypo-methylated in DP cells as a result of shock wave exposure, we were able to identify cilengitide, an ITGAV inhibitor, which was capable of abrogating ossification in both human DP cells and BM-MSCs. We believe that DP cells are an excellent *in vitro* model to study inappropriate ossification, or blast trauma-induced HO, and as shown here, our model system can be used to identify therapeutics to prevent disease onset. Our data indicate that cilengitide is an effective inhibitor of HO *in vitro*, however, ultimately we seek to translate these findings and demonstrate prevention of HO *in vivo*.

The following are the supplementary data related to this article.

Supplementary material Image 1

Movie S1 Live cell imaging of shock wave response in DP cells in GM and OM. Movie S1
