# Supplementary material for: Demethylation of ITGAV accelerates osteogenic differentiation in a blast-induced heterotopic ossification in vitro cell culture model
Source: Bone. 2018 Dec;117:149–60. doi: 10.1016/j.bone.2018.09.008 (PMC6218666; doi:10.1016/j.bone.2018.09.008)
Supplement: Supplementary file 1 — Supplementary material [file mmc1.docx]

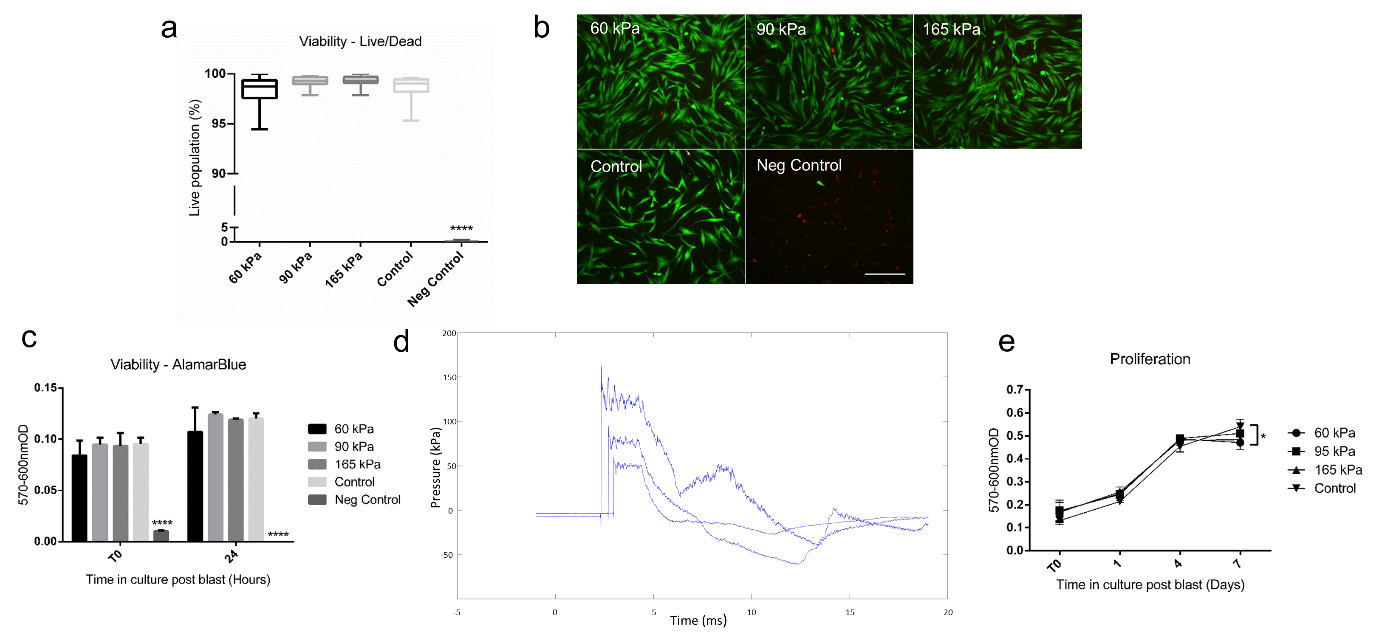


**Fig. S1. Shock wave viability analysis.** (**a**) Quantification of live dead images showing no significant increase in cell death in human DP cells following shock wave exposure compared to the control. Each box plot shows the upper and lower quartiles with Tukey whisker. n = 12, **** = p ≤ 0.0001, one way ANOVA plus Tukey’s multiple comparisons test. (**b**) Representative fluorescent images from live dead analysis. Green shows live viable cells, whilst red signifies dead cells. Scale bar = 300μm. (**c**) Alamarblue analysis at both T0 and 24 hours post shock wave exposure showed no significant decrease to cell viability compared to the control in human DP cells. Each bar represents the mean ± 1 SD, n = 3. **** = p ≤ 0.0001, two way ANOVA plus Tukey’s multiple comparisons test. (**d**) An approximation of a Friedlander wave observed at the cell loading location on the shock tube at the three different firing pressures used in viability testing. (**e**) Proliferation data from human DP cells showing no clear effect of the shock wave on proliferative ability compared to cells in control media (GM). At 7 days cells exposed to a 60 kPa shock wave had proliferated less compared to the control, however this effect was not present in other higher kPa shock wave treated conditions. Data represent the mean ± 1 SD, n = 3. * = ≤ 0.05, two way ANOVA plus Tukey’s multiple comparisons test.


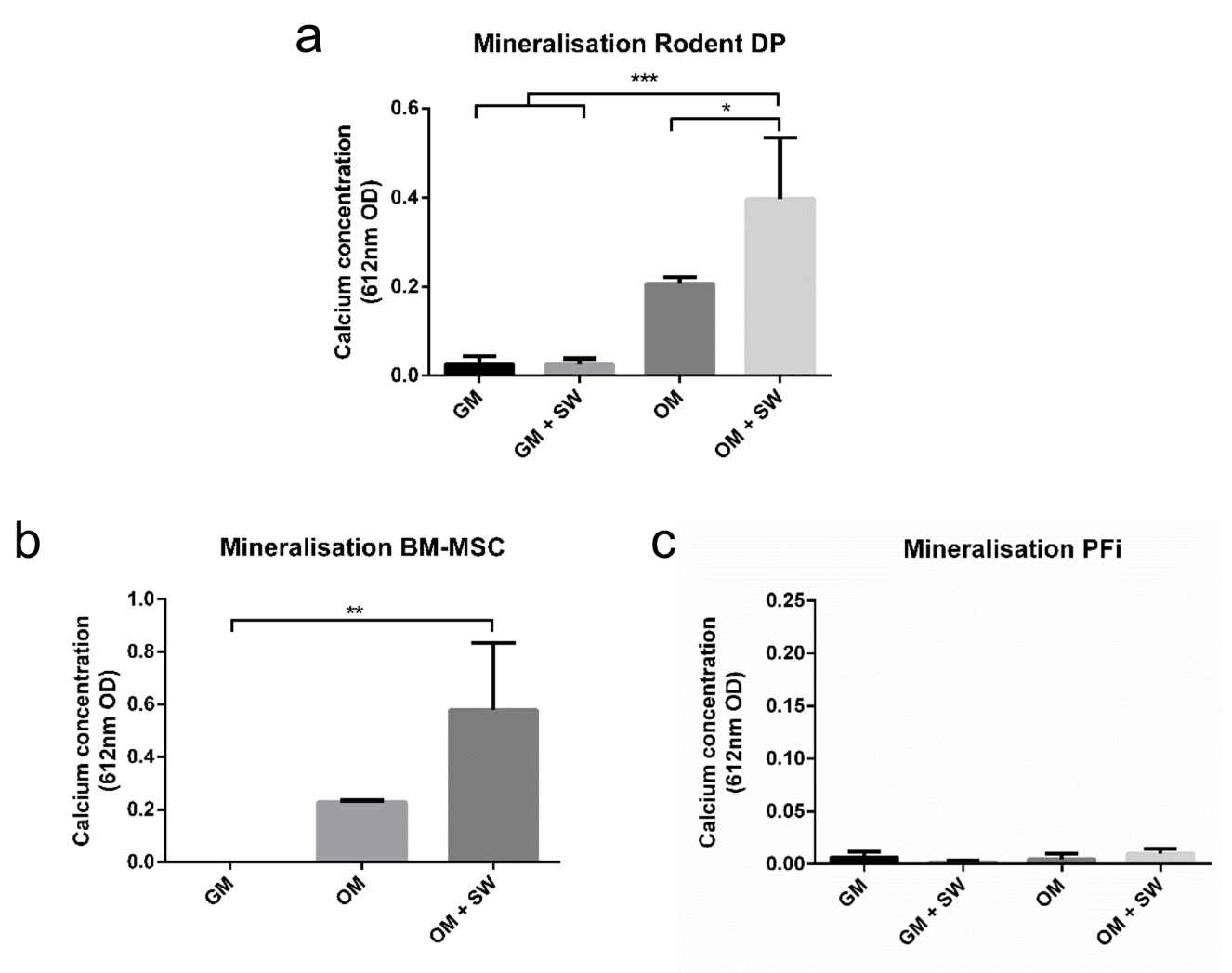


**Fig. S2. Shock wave-induced osteogenesis in BM-MSC, PFI and rodent DP**: Mineralisation data showing calcium concentration for (**a**) rodent DP cells at 14 days, (**b**) human BM-MSCs at 15 days, and lastly (**c**) human dermal PFi at 8 days. The synergistic effect of the shock wave and OM is present in other cells types such as BM-MSC and rodent DP. However, DP sister cell type, dermal PFi did not appear to be affected by either OM or OM+SW. Each bar represents the mean ± 1 SD, n = 3. * = p ≤ 0.05, ** = p ≤ 0.01, *** = p ≤ 0.001, one way ANOVA plus Tukey’s multiple comparisons test.


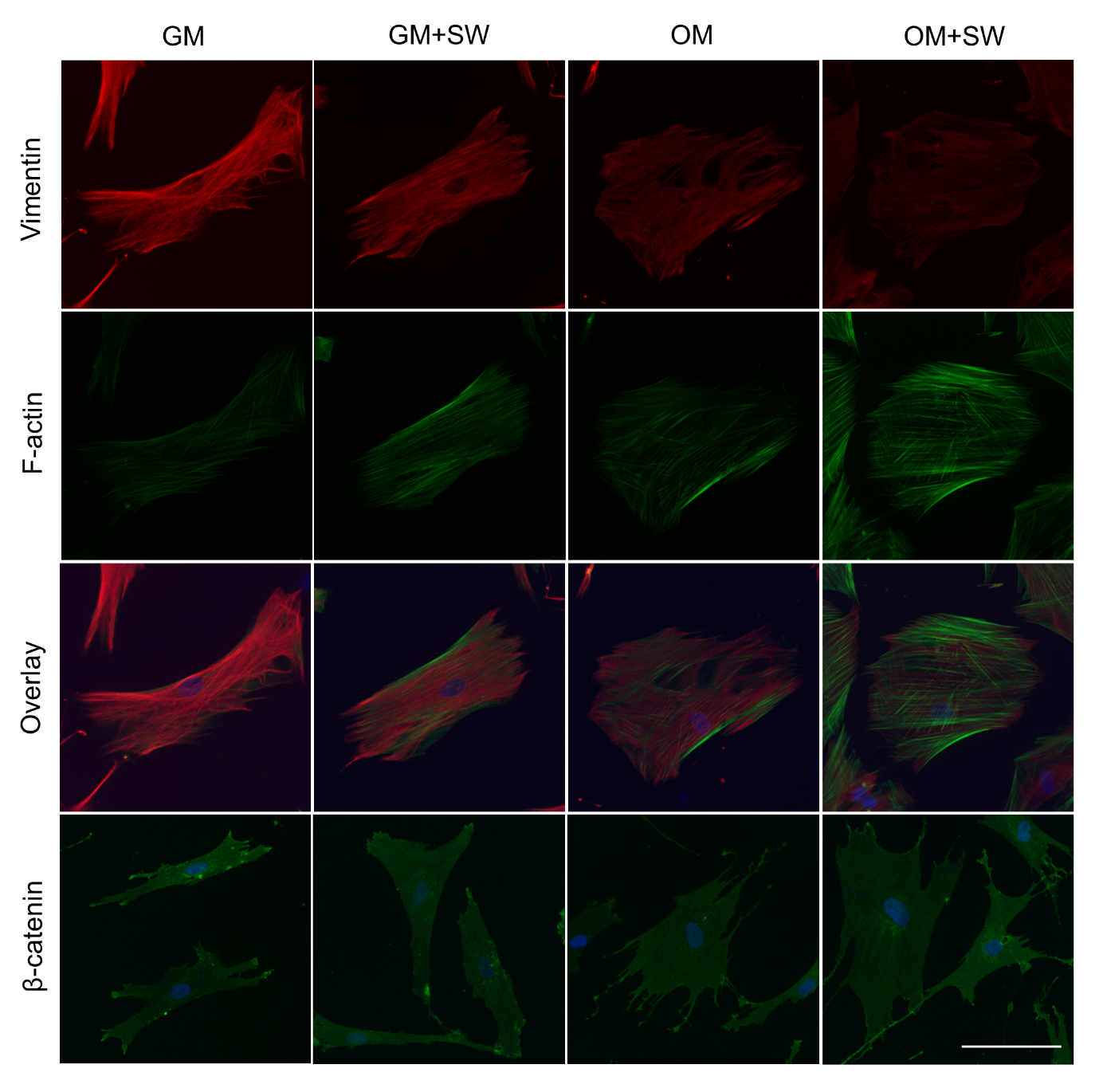


**Fig. S3: Cell morphological immunofluorescence analysis.** Cytoskeletal markers f-actin and vimentin, as well as osteogenic associated β-catenin were imaged at 24 hours. Cell shape was clearly different between cells in GM compared to OM, with cells cultured in OM appearing larger and more spread. The shock wave did not appear to have a clear effect on vimentin, however vimentin was strongly expressed in samples cultured in GM when compared to those observed in OM, which appeared to lose their expression. In contrast, f-actin fibres appeared thicker and more robust in shock wave exposed samples compared to their media controls, with the strongest f-actin presence observed in OM+SW cells. Β-catenin was detected throughout the cell and no clear translocation into the nucleus was observed in OM samples compared to GM. Vimentin presented as red, counterstained with f-actin in green and cell nuclei in blue using DAPI. Β-catenin shown as green counterstained with DAPI as blue. Scale bar = 100 μm


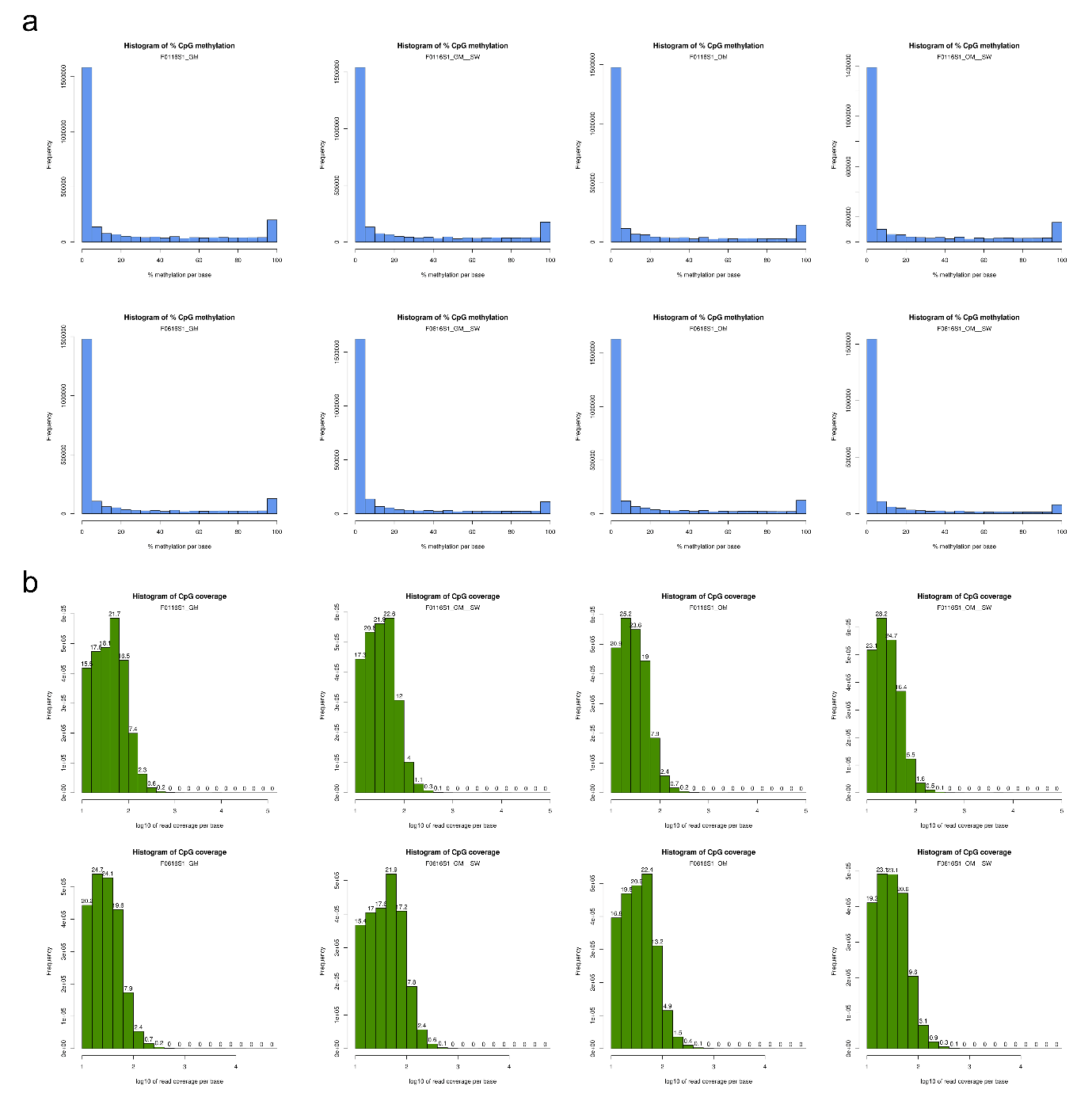


**Fig. S4. RRBS coverage and distribution.** (**a**) Percent methylation distribution per base for each sample. (**b**) CpG coverage statistic for each sample.


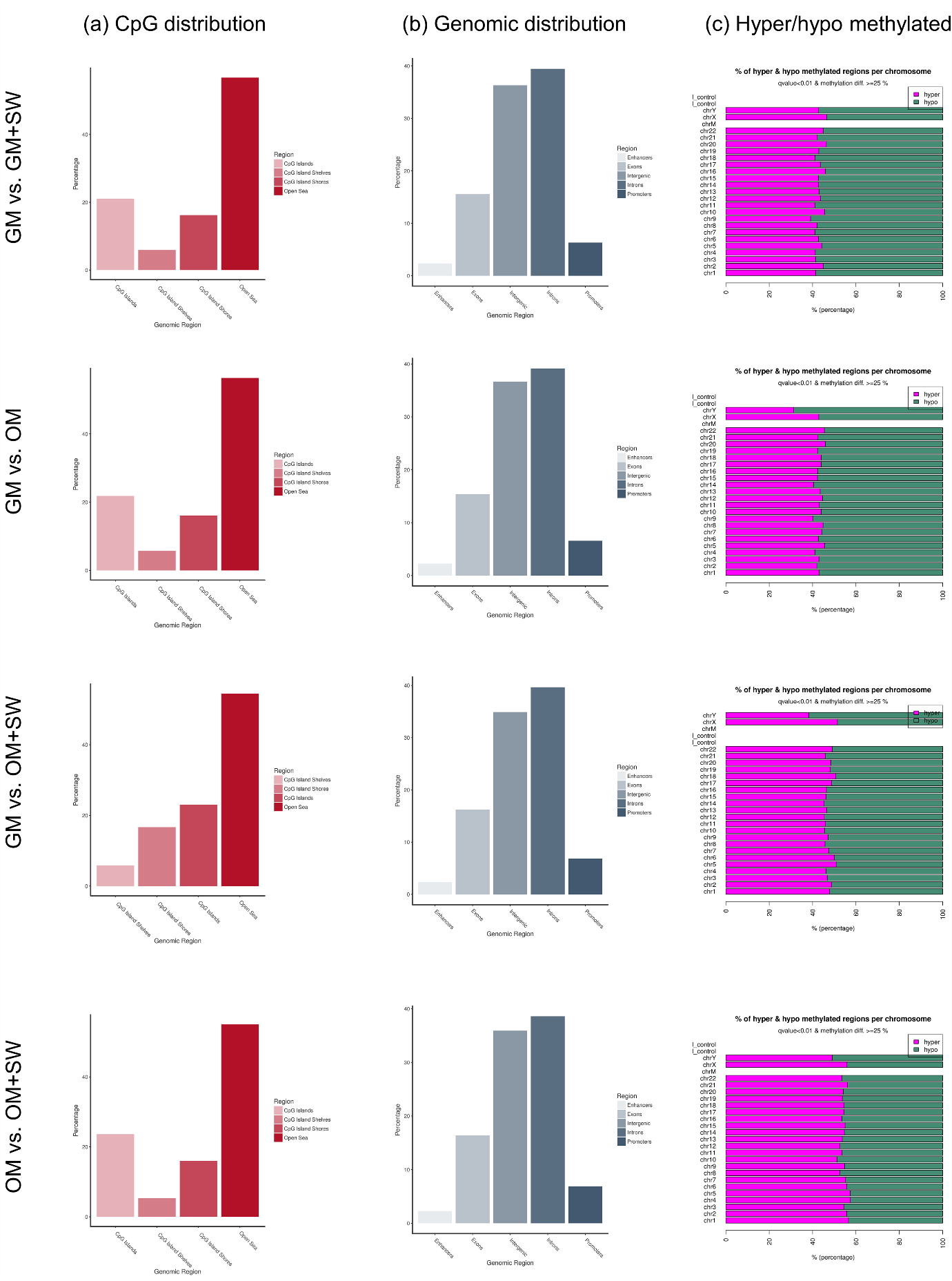


**Fig. S5. RRBS genomic distributions.** (**a**) Genomic distribution of differentially methylated CpGs in each of the performed comparisons, including GM vs GM+SW, GM vs. OM, GM vs. OM+SW and OM vs. OM+SW. Regions include CpG Island Shelves, CpG Island Shores, CpG Islands and Open Sea. (**b**) Distribution of differentially methylated CpGs throughout the genome including enhances, exons, intergenic, introns and promoters. (**c**) Differential methylation per chromosome for each condition. Shown as percentage of hyper and hypo methylated regions per chromosome.


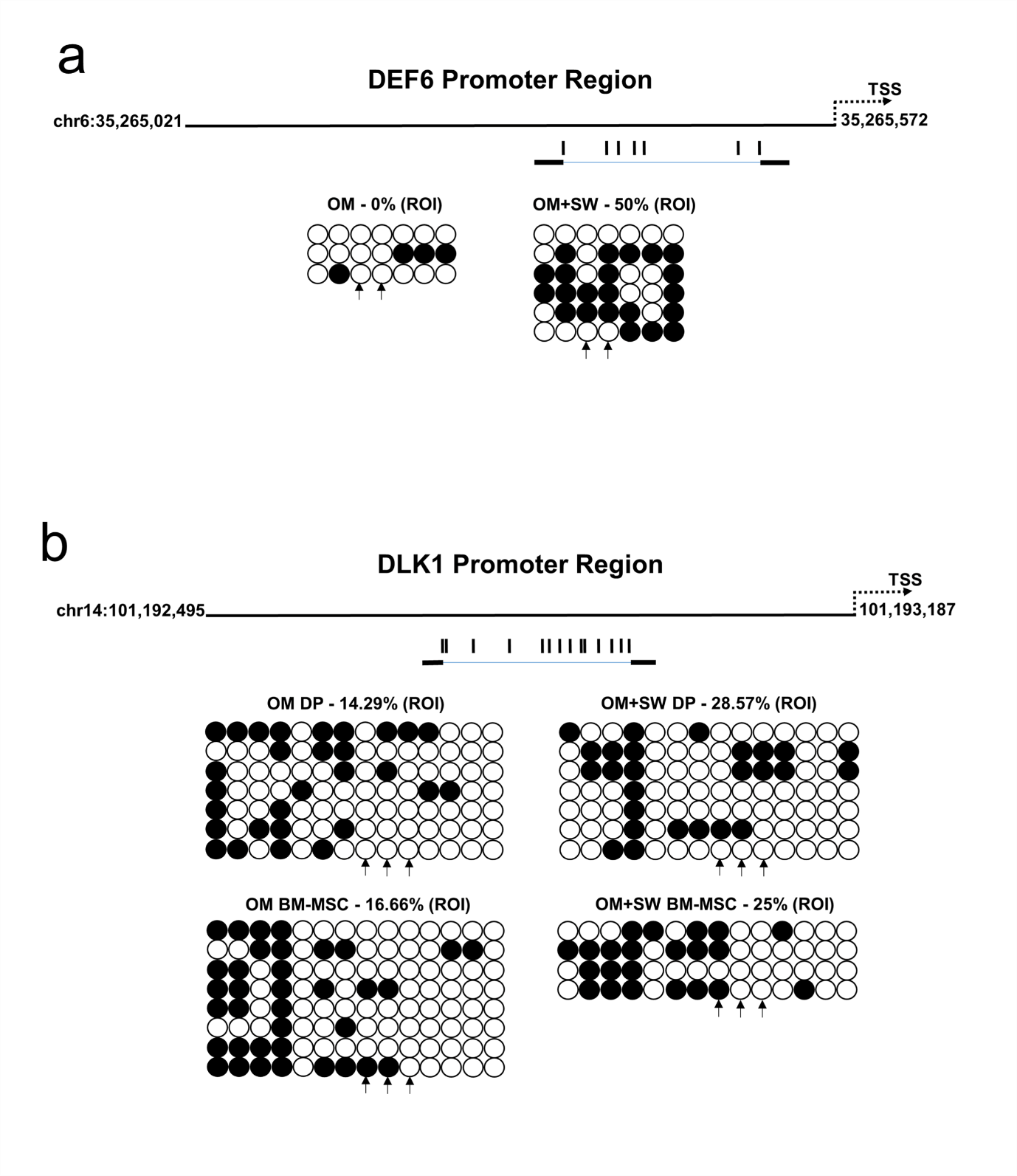


Fig. S6. Bisulfite Sequencing. (**a** and **b)** Bisulfite sequencing of a region of interest (ROI) located in the promoter region of DEF6 in human DP cells and DLK1 in human DP and BM-MSCs in OM and OM+SW conditions (filled circles represent a methylated CpG site while open circles represent no methylation). Arrows indicate CpGs that fall within the ROI identified in RRBS analysis.


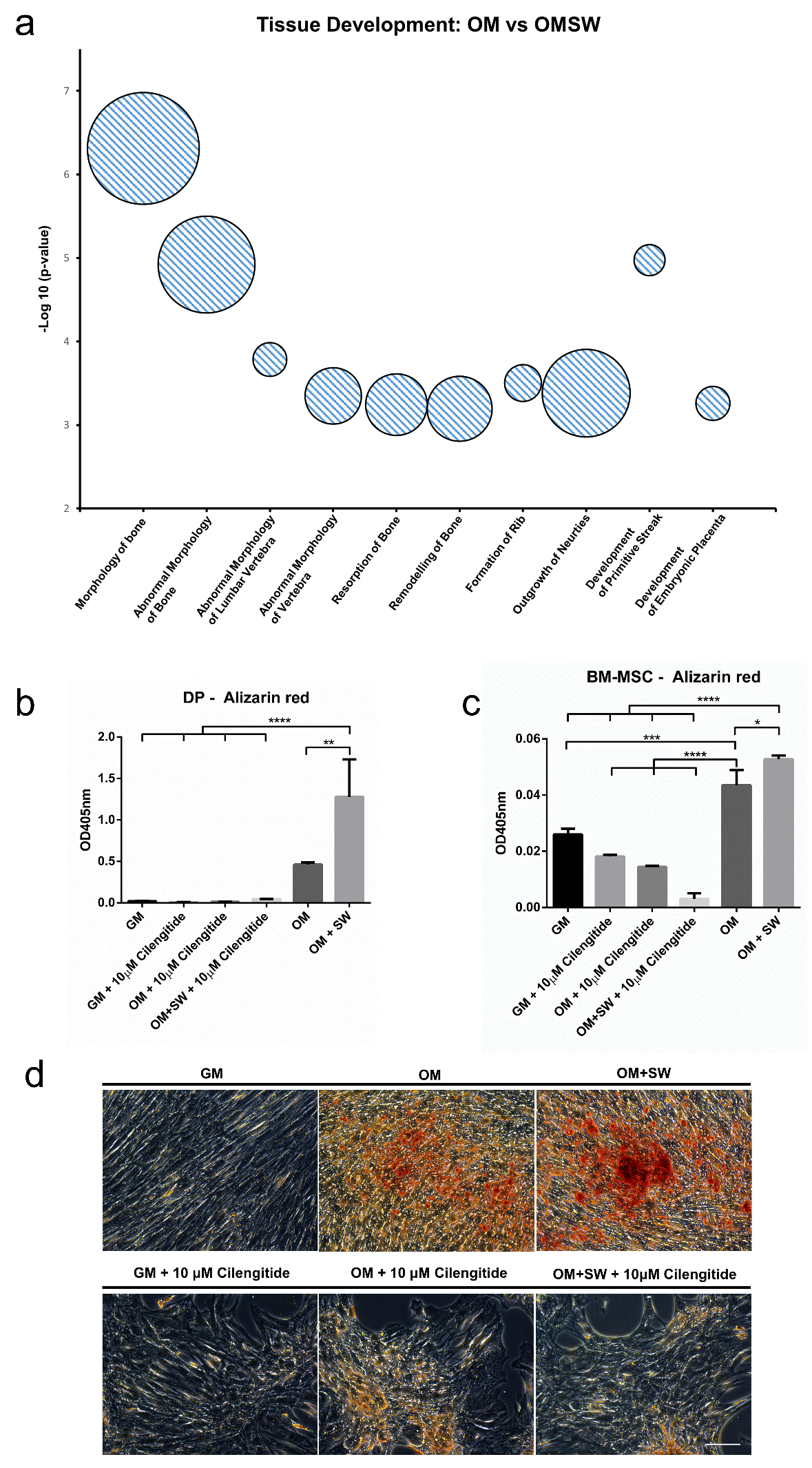


**Fig. S7. Cilengitide can abrogate mineral deposition.** (**a**) Visualisation of the top 10 annotations of the tissue development function for the OM vs. OM+SW condition, which shows an enrichment towards bone formation and development. The size of each bubble represents the number of genes in that category. All p values generated in IPA using Fisher’s exact test. (**b**) Quantification of alizarin red staining of human DP cells after 14 days in culture. The presence of cilengitide has abrogated mineral deposition. Each bar represents the mean ± SD, n = 3. ** = p ≤ 0.01, **** = p ≤ 0.0001, one way ANOVA plus Tukey’s multiple comparisons test. (**c**) Quantification of alizarin red staining of human BM-MSCs after 14 days in culture. The presence of cilengitide prevented the onset of mineral deposition, as signals shown are below the GM negative control. Each bar represents the mean ± SD, n = 3. * = p ≤ 0.05, *** = p ≤ 0.001, **** = p ≤ 0.0001, one way ANOVA plus Tukey’s multiple comparisons test. (**d**) Representative images of human BM-MSC alizarin red staining after 14 days in culture. Positive red staining indicates the presence of mineral deposition. Scale bar = 100μm.

**Table. S1. Primer sequences used in study.** GAPDH, DLX5 and RUNX2 taken from Farshdousti Hagh et al^50^. Other primers designed against sequences in the UCSC database.

| **Primer name** | **Sequence (5’ to 3’)** | **Annealing temperature (°C)** |
| --- | --- | --- |
| RUNX2-F | CCCCACGACAACCGCACCAT | 60 |
| RUNX2-R | CGCTCCGGCCCACAAATCTC |  |
| DLX5-F | ACCAACCAGCCAGAGAAAGA | 60 |
| DLX5-R | TCTCCCCGTTTTTCATGATC |  |
| GAPDH-F | CGTCTTCACCACCATGGAGA | 60 |
| GAPDH-R | CGGCCATCACGCCACAGTTT |  |
| MAP2K2-F | AGGTCCTGCACGAATGCAA | 60 |
| MAP2K2-R | CGTCCATGTGTTCCATGCAA |  |
| DLK1-F | GCACTGTGGGTATCGTCTTCC | 60 |
| DLK1-R | CTCCCCGCTGTTGTACTGAA |  |
| KCP-F | CTGCGTTTGGGAATAGCTGG | 60 |
| KCP-R | ACACAGGTCATACACACAGG |  |
| CEBPB-F | AGAGCAAGGCCAAGAAGACC | 60 |
| CEBPB-R | GCTGCTCCACCTTCTTCTGC |  |
| DEF6-F | ATGAACCGGCTGATGCATCC | 60 |
| DEF6-R | CTGCTGCTCATTGCTGTTGG |  |
| ITGAV-F | AAGTCCCATCAGTGGTTTGG | 60 |
| ITGAV-R | AATCCCTGTCCATCAGCATC |  |
| ITGAV-Bisulfite F | GTTTTTGTTTTTAAGGTTTTTTTT | 51 |
| ITGAV-Bisulfite R | ACCTTATTCCCAATTTATTAACTAC |  |
| DLK1-Bisulfite F | TTATGGTTAGGGGTATAGGGG | 58 |
| DLK1-Bisulfite R | CATACAAACCTACCTAAAACAAATC |  |
| DEF6-Bisulfite F | GGAGTTTTGGAAGGTTTTAGAAGTT | 61 |
| DEF6-Bisulfite R | AAAACACCACCTACCTACACATTAC |  |

**Table. S2. Summary of RRBS statistics**

| **Sample Name** | **Total Reads** | **Uniquely aligned** | **Mapping efficiency (%)** | **CpGs detected** | **Average coverage** |
| --- | --- | --- | --- | --- | --- |
| F0116S1_GM | 65,318,735 | 44,719,914 | 68.46 | 4,034,858 | 38 |
| F0116S1_GM_SW | 53,447,896 | 36,044,316 | 67.44 | 3,960,419 | 30 |
| F0116S1_OM | 41,611,575 | 28,536,148 | 68.58 | 3,794,545 | 25 |
| F0116S1_OM_SW | 37,371,128 | 25,098,417 | 67.16 | 3,759,369 | 22 |
| F0616S1_GM | 35,295,720 | 25,683,646 | 72.77 | 3,602,676 | 25 |
| F0616S1_GM_SW | 47,085,304 | 35,879,318 | 76.20 | 3,685,041 | 37 |
| F0616S1_OM | 43,636,231 | 32,923,488 | 75.45 | 3,588,175 | 33 |
| F0616S1_OM_SW | 33,488,631 | 25,046,607 | 74.79 | 3,447,296 | 27 |

**Table. S3. Distribution of hypo and hyper-methylated gene promoters.**

|  | **Number of hypomethylated gene promoters** | **Number of hypermethylated gene promoters** |
| --- | --- | --- |
| GM vs GM+SW | 538 | 364 |
| GM vs OM | 568 | 453 |
| GM vs OM+SW | 516 | 504 |
| OM vs. OM+SW | 438 | 525 |
